# E3 ubiquitin ligase, RNF139, inhibits the progression of tongue cancer

**DOI:** 10.1186/s12885-017-3438-7

**Published:** 2017-06-29

**Authors:** Lina Wang, Wei Yin, Chun Shi

**Affiliations:** 1 The State Key Laboratory Breeding Base of Basic Science of Stomatology (Hubei-MOST) & Key Laboratory of Oral Biomedicine Ministry of Education, School & Hospital of Stomatology, Wuhan University, 237 Luoyu road, Wuhan, 430079 China; 2 Department of Endodontics, College of Stomatology, Dalian Medical University, Dalian, 116044 China

**Keywords:** RNF139, Cell viability, Invasion, Tongue cancer, SCC25 cells

## Abstract

**Background:**

Tongue cancer is still one of the leading causes of mortality around the world. Recently, the ubiquitin system has been established as a critical modulator of tumors. In order to find the oral cancer related E3 ubiquitin ligases, we screened the human E3 ubiquitin ligase library and found that RING finger protein 139 (RNF139) regulated the biological behavior of tongue cancer cells.

**Methods:**

MTT assay was used to analyze the cell viability changes of tongue cancer SCC9 and SCC25 cells caused by RNF139. The invasion ability of SCC9 and SCC25 cells with or without the knockdown of RNF139 was evaluated through transwell assay. The immunoblotting was recruited to determine the expression level of RNF139 in human tongue cancer tissues and para-carcinoma tissues. The effect of RNF139 on tumorigenicity of tongue cancer cells was analyzed by xenograft model on immunodeficient Balb/c nude mice.

**Results:**

Overexpression of RNF139 inhibits the viability of tongue cancer cells since day 2. The colony formation ability of SCC9 and SCC25 cells was also decreased with the overexpression of RNF139. Knockdown of RNF139 significantly promoted the invasion ability of SCC9 and SCC25 cells. Furthermore, knockdown of RNF139 also induced the activation of AKT signaling pathway. While human tongue cancer tissues had low expression of RNF139. In nude mice, knockdown of RNF139 promoted the tumorigenicity of the SCC25 cells.

**Conclusions:**

Our data establish a role for RNF139 in regulating the progression of tongue cancer.

## Background

Tongue cancer is one of the leading cancers in prevalence and around 16,400 new American cases are estimated in 2017 [1]. The dysfunction of P53 signaling pathway, phospho-inositide-3-kinase (PI3K)/v-akt murine thymoma viral oncogene homolog (AKT) signaling pathway as well as transforming growth factor-β (TGFβ) signaling pathway plays a critical role in the carcinogenesis of tongue cancer. The activity of these signaling pathways is regulated by post-translational modification.

Ubiquitination is one of the post-translational modification which involves in several cellular activity, including gene transcription, cell-cycle control, DNA repair and protein degradation [2–5]. It is mediated by the sequential participated enzymes, E1 ubiquitin activating enzymes, E2 ubiquitin conjugating enzymes, and E3 ubiquitin ligases [6, 7]. Several E3 ubiquitin ligases have been confirmed to participate in the pathogenesis of cancers [8–11]. Although the role of E3 ubiquitin ligases is still not well-understood, it is continuously being discovered [12–14].

In order to find the oral cancer related E3 ubiquitin ligases, we screened the human E3 ubiquitin ligase library [12] and found that RING finger protein 139 (RNF139) regulated the proliferation of tongue cancer cells. In this study, we further analyzed the role of RNF139 on the development of tongue cancer.

## Methods

### Reagents and antibodies

Lipofectamine 2000 (Life Technologies), 3-(4,5-dimethylthiazol-2-yl)-2,5-diphenyltetrazolium bromide (MTT) (Antgene), mouse antibodies against β-actin (Sigma) and RNF139 (Santa Cruz); rabbit polyclonal antibodies against AKT (CST), phospho-AKT (308 and 473) (CST), phospho-FoxO1 (CST), phospho-GSK3β (CST), and phospho-mTOR (CST) were purchased from the indicated manufacturers. SCC9 (CRL 1629™), SCC25 (CRL 1628™) and HEK293 (CRL 1573™) cells were obtained from ATCC.

### Cell culture, plasmid and RNAi construction

Human tongue cancer SCC9 and SCC25 cells were maintained in DMEM medium with the supplement of 10% FBS and 1% Penicillin-Streptomycin. Mammalian expression plasmids for the RNF139 and RNF139-RNAi were constructed according to the instructions of molecular cloning: a laboratory manual.

### The stable RNF139 overexpression/knockdown SCC9 and SCC25 cells

The protocol was the same with our previous report [11]. In brief, the RNF139 or RNF139-RNAi retrovirus was packaged in the HEK293T cells and incubated with SCC9 and SCC25 cells. The retrovirus infected SCC9 and SCC25 cells were treated with puromycin (0.5 mg/ml) for 7 days before the following experiments.

### Cell viability assay

The stable RNF139 overexpression/knockdown SCC9 and SCC25 cells (2 × 10^3^) were seeded on the 96 well plates. MTT (5 μg/ml) was used and incubated at 37 °C for 4 h. Then the cells were incubated with Dimethyl sulfoxide (DMSO) for 30 min. The cell viability was determined by microplate reader at day 2, 4 and 6.

### Cell invasion assay

The cell invasion assay was performed with the same procedure [11]. Briefly, after coating with the matrigel basement membrane matrix, the upper chamber of the transwell plate was seeded with the stable RNF139 overexpression/knockdown SCC9 and SCC25 cells with serum-free DMEM medium. The complete DMEM medium was added into the bottom chamber. They were incubated at 37 °C for 24 h. After fixing and staining, the transwell membrane was graphed by Olympus IX71 light microscope.

### Colony formation assay

The stable RNF139 overexpression SCC9 and SCC25 cells were cultured in 10-cm cell dishes for 18 days. The cell culture medium was changed every other two days. The cell colonies were stained with crystal violet and quantified with ImageJ software.

### Immunoblotting analysis

Cells were lysed with the lysis buffer (Nonidet P-40 buffer). Protein were seperated with the polyacrylamide gel electrophoresis and transferred into polyvinylidene membrane. The membrane was immunoblotted with the correspondent antibodies and developed with the ECL reagent.

### Xenograft model

Eight-week-old male athymic immunodeficient Balb/c nude mice were purchased from Shanghai Laboratory Animal Center. The stable RNF139-knockdown SCC25 (5 × 10^7^) cells were injected subcutaneously into the flank. Tumor diameters were recorded every 4 days. Specimens were harvested at 40 days.

### Patients and specimen collection

The tumor tissues of tongue cancer patients were collected from Jan. 2016 to Sep. 2016. None of them received any anticancer therapies before surgery. The diagnosis was based on the pathologically analysis. The total protein was extracted with the Minute TM Total Protein Extraction Kit (Inventbiotech).

### Statistics

The tumor volume of mice xenograft was analyzed by two-way ANOVA. One-way ANOVA was used to analyze the results of cell viability assay, cell invasion assays and colony formation assays. All data were analyzed by the SPSS package for Windows (Version 18.0, Chicago, IL). The statistically significant refers to the *P* value <0.05.

## Results

### RNF139 inhibits the viability of tongue cancer cells

**Fig. 1:**
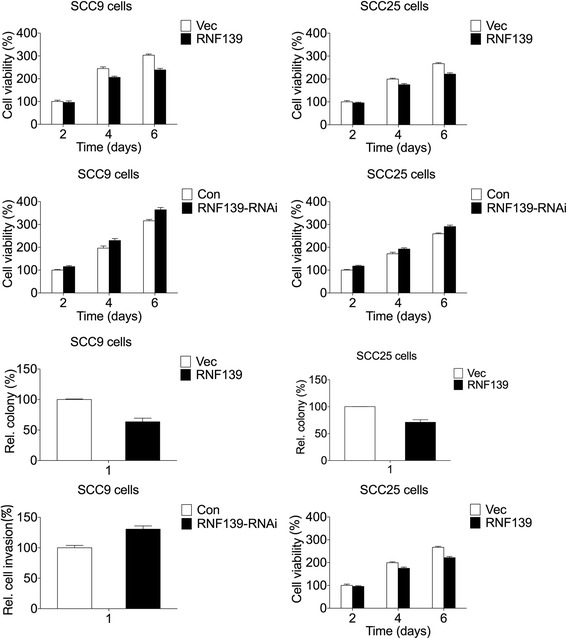
RNF139 regulates the viability, colony formation and invasion of tongue cancer SCC9 and SCC25 cells. **a** Overexpression of RNF139 inhibits the viability of SCC9 and SCC25 cells. *N* = 6. **b** Knockdown of RNF139 promotes the viability of SCC9 and SCC25 cells. *N* = 6. **c** Overexpression of RNF139 inhibited the colony formation ability of SCC9 and SCC25 cells. *N* = 3. **d** Knockdown of RNF139 promoted the invasion of SCC9 and SCC25 cells. The gel/blots which indicates the efficiency of RNF139 overexpression were the same gel/blots in (**a**) and (**c**). The gel/blots which indicates the efficiency of RNF139 knockdown were the same gel/blots in (**b**) and (**d**). *N* = 3. *:*P* < 0.05, **:*P* < 0.01

In order to elaborate the role of RNF139 in regulating the biological behavior of tongue cancer, we analyzed the viability changes of tongue cancer cells, SCC9 and SCC25 cells, induced by RNF139. The results suggested that overexpression of RNF139 significantly inhibited the viability of SCC9 and SCC25 cells (Fig. 1a). While knockdown RNF139 had the opposite effects (Fig. 1b). Then we further analyzed the effect of RNF139 on colony formation ability of SCC9 and SCC25 cells. As shown in Fig. 1c, the number of cell colony was also decreased with the overexpression of RNF139.

### RNF139 regulates the invasion of tongue cancer cells

We next investigated the functions of RNF139 on cell invasion. The transwell assay was used to analyze the invasion changes of tongue cancer cells. Knockdown of RNF139 significantly promoted the invasion ability of SCC9 and SCC25 cells (Fig. 1d). Taken together, these data suggest that RNF139 inhibits cell viability and invasion of tongue cancer SCC9 and SCC25 cells.

### Human tongue cancer tissues had low expression of RNF139

**Fig. 2:**
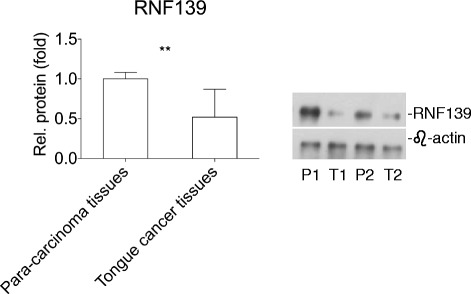
Human tongue cancer tissues had low expression of RNF139. **a** Compared to the para-carcinoma tissues, tongue cancer tissues had low level of RNF139 protein in tongue cancer patients (*N* = 23). **b** The immunoblotting analysis of RNF139 expression in para-carcinoma tissues and tongue cancer tissues of five tongue cancer patients. P: para-carcinoma tissues, T: tongue cancer tissues

To analyze the role of RNF139 in human lung cancers, we detected the protein level of RNF139 in 23 tongue cancer patients’ tumor tissues through western blotting. Most of these patients were 51–70 years old. Squamous cell carcinoma accounted for 91.30% (21 out of 23). The protein level of RNF139 was significantly decreased in the tongue cancer tissues in comparison to para-carcinoma tissues (Fig. 2a&b).

### RNF139 promotes activation of AKT1

**Fig. 3:**
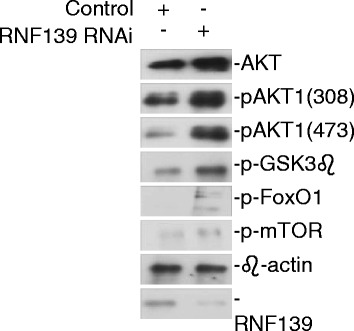
Knockdown of RNF139 promotes activation of AKT1 signaling pathway in SCC9 and SCC25 cells. The blots of RNF139 and β-actin were the same blots in Fig. 1b and d

After determining the correlation between RNF139 and tongue cancer, we further analyze the regulating mechanism of RNF139 on tongue cancer. We screened the expression changes of critical components in PI3K-AKT, JAK-STAT, p53 and MAPK signaling pathway. The AKT signaling pathway had evident changes with the knockdown of RNF139. As shown in Fig. 3, knockdown of RNF139 significantly potentiated the phosphorylation of AKT1 at Ser308 and Ser473, which is the hall mark of AKT1 activation [28]. Meanwhile, knockdown of RNF139 also induced the activation of downstream molecules of AKT1, such as mTOR, FoxO1 and GSK3β in SCC9 and SCC25 cells.

### Knockdown of RNF139 promoted the growth of the tumor xenografts

**Fig. 4:**
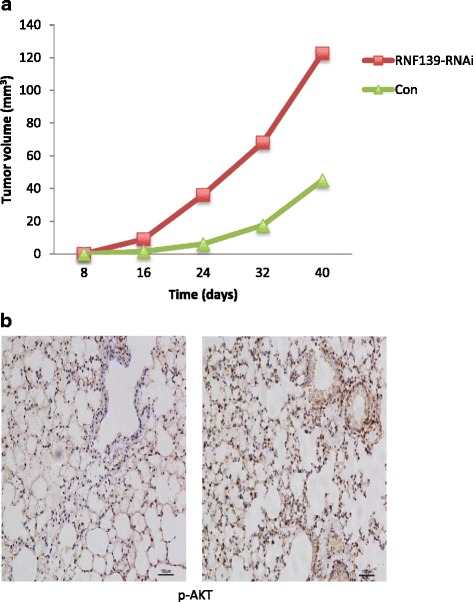
Knockdown of promoted the tumorigenicity of the SCC25 cells. **a** The growth curve of xenograft tumor model of nude mice which was originated from the SCC25 cells with or without knockdown of RNF139. **b** The expression of pAKT1 was increased in the xenografts which was originated from with the RNF139 stable knockdown SCC25 cells

Next, we performed in vivo xenograft analysis by subcutaneously injecting stable RNF139 knockdown SCC25 cells into nude mice to further analyze the function of RNF139. As shown in Fig. 4a, knockdown of RNF139 markedly promoted the volumes of the tumor xenografts. The histological analysis indicated that the expression of pAKT1 was strongly increased in the SCC25 cells induced xenografts with the knockdown of RNF139 (Fig. 4b).

## Discussion

Despite advances made in detection and diagnosis as well as treatments in the past ten years, tongue cancer is still one of the leading causes of mortality around the world [12]. The body of the tongue has abundant lymphatic and blood vessel and frequent movement which promotes the metastasis of tongue cancer. The impressive progresses achieved in tumor biology make us realize that each tumor has unique character and should receive corresponding precision treatment. In the past decades, patients with breast and lung cancer already benefited from the personalized treatment which aimed to treat patients according to their own molecular characteristics [15, 16]. In order to search the candidate treatment target for tongue cancer, we focused on the E3 ubiquitin ligase and screened the human E3 ubiquitin ligase library. RNF139 as was one of the candidates.

Ubiquitin is a widely existed protein which can be found in all eukaryotic cells. It consists of 76 amino acids with an exposed C-terminal. The isopeptide bond connection between the carboxyl-terminal glycine residue of ubiquitin and an internal K residue or the amino-terminal methionine (M1) of another ubiquitin forms the polyubiquitin chains. Ubiquitination is indispensable for several biological processes. Several E3 ubiquitin ligases have been confirmed to be related with initiation and progression of tumor [12, 13].

E3 ubiquitin ligases mainly include RING and HECT type E3 ubiquitin ligases. The multimembrane-spanning protein RNF139 belongs to the RING type E3 ubiquitin ligases and has a RING-H2 domain with E3 ubiquitin ligase activity at the COOH-terminal. It locates in the endoplasmic reticulum (ER) and transfers the ubiquitin from the E2 ubiquitin conjugating enzymes to substrate. Previous study suggested that RNF139 could utilize several E2 ubiquitin conjugating enzymes for ubiquitylation. RNF139 ubiquitinated and degraded the antioxidant enzyme heme oxygenase-1 (HO-1) and suppressed HO-1-induced cancer cell growth, migration and invasion [17]. In this study, we contributed to add another evidence that RNF139 was a antioncogene protein. We demonstrated that RNF139 was a suppressor of tongue cancer SCC9 and SCC25 cells growth and invasion. Knockdown of RNF139 promoted the tumorigenicity of the SCC25 cells. Although we did not find the direct substrate of it, we observed the significant changes in AKT signaling pathway which was induced by RNF139. Furthermore, we determined the low expression of RNF139 in human tongue cancer tissues. These results support a tumor suppressor role for RNF139 in tongue cancer.

## Conclusions

Our data clearly establish a correlation between RNF139 and tongue cancer. Of clinical relevance is the fact that our results contribute to the new molecule treatment targets for tongue cancer.

## Abbreviations

AKT: v-akt murine thymoma viral oncogene homolog
DMSO: Dimethyl sulfoxide
ER: Endoplasmic reticulum
HO-1: Heme oxygenase-1
PI3K: Phospho-inositide-3-kinase
RNF139: RING finger protein 139
TGFβ: Transforming growth factor-β
